# Cephalanthus Occidentalis (Kephalahead Anthos Flower) Globe Flower

**Published:** 1871-10

**Authors:** R. C. Word

**Affiliations:** Tunnel Hill, Ga.


### CEPHALANTHUS OCCIDENTALIS (*Kephalahead Anthos Flower*)–GLOBE FLOWER.

BY R. C. WORD, M. D., TUNNEL HILL, GA.

The above plant, known throughout many portions of the Southern States, under the several names of *Button Bush*, *Elbow Weed*, *Globe Flower*, *etc.*, is, we think, likely to prove a valuable addition to the list of our indigenous remedies.

It is very abundant in Upper Georgia, particularly in this Section (Whitfield County). It is a thick shrubbery bush, with roots 12 to 18 inches long, and the size of the finger or larger. It grows in low, marshy places along the branches and in the meadows. The bush varies in size from a foot to five or six feet in height; first a single stalk, which spreads and throws out additional stems. Many of the old ones die each year and new ones spring up, making a thick, dense cluster of dead and living twigs, which, being very crooked and often bent at right-angles, has, we suppose, given rise to the name *Elbow Weed.* The leaves grow in triplets, obovate, green, smoothe upon the upper surface, and ordinarily, about 3 or 4 inches long, though sometimes double this size. The flowers or *buttons* make their appearance in mid-summer, upon three foot stalks corresponding in number to the leaves, and coming out immediately above them; at first, small, hard spherical bodies, which gradually enlarge to the size of a walnut, and then flowering on its entire circumference, constitutes a beautiful *Globe Flower.*

The root is the part used as a medicine. It has a pleasant bitter taste, resembling that of Sarsaparilla. The bark of the stem is more bitter than the root, and is very astringent. The leaf combines an acid taste with the bitter astringent property of the bark.

Many years ago, while engaged in the practice of medicine in the county of Cass, my attention was frequently drawn to the *Button Bush*, as a domestic remedy in *chronic coughs and catarrhal affections;* but it was not until I had been made aware of the cure of a long and obstinate case of Chronic Bronchitis, in which the expectoration was profuse, and the patient worn down and emaciated to the last degree, that my attention was sufficiently aroused to give the remedy a trial in practice. This I first did in a case of unmistakable Phthisis Pulmoralis, with marked benefit to the patient—using a strong tea of the root. Again, in 1868, I used it in the latter stages of a hopeless case, with much relief to the cough and other distressing symptoms. This patient was greatly troubled with dyspepsia, consequent upon a torpid condition of the liver, which was relieved, in a marked degree, by the *Button Wood*, thus disclosing a valuable property of the plant, not previously ascribed to it—that of imparting tone to the liver and digestive organs. As corroborative of this feature of the remedy, we have recently learned that in a certain neighborhood in this county, it is called "dyspepsia root," the fact being related to me by an old gentleman, a dyspeptic, who carries a piece of the root in his pocket, and chews it, swallowing the juice, as a means of relief to a troublesome indigestion.

Not many months ago we prescribed the Button Wood tea to an old lady, aged 60 years, laboring under a chronic bronchial cough of about four months duration. The cough was deep, harsh and very exhausting, particularly at night, and the expectoration rather scant and difficult. This case was wholly relieved in about two weeks' time, and the patient rapidly recovered her strength.

Another fact occurs in connection with this medicine, which we will mention,—it is the case of Mrs. I., of Kingston, Georgia, a confirmed Asthmatic. Her case is dependent upon organic lesion of the heart, and therefore incurable. Yet she has found great relief from the Button Wood tea, in the severe paroxysms to which she is subject.

We are unable to explain the *modus operandi* of this medicine, and our observations are yet too limited to give a full and satisfactory account of its properties.

It is evidently *tonic*, *alterative*, *expectorant*, slightly *laxative*, and in large doses, *nauseating*. Its purgative or laxative tendency seems to be directed to the duodenum and upper bowels, gently stimulating the liver, and invigorating the digestive apparatus. It exerts a particularly soothing influence upon the bronchial mucous membrane, and is specially adapted to patients laboring under pectoral affections in connection with hepatic derangement. It may be supposed that it benefits the lungs indirectly by its invigorating action upon the digestive and nutritive functions. It is not improbable that the results are due in some degree to this fact. Yet there can be no doubt that there is an independent action exerted upon the lungs and air passages; as under its influence the mucous secretion is speedily modified and softened, the expectoration is made easy, and the cough rapidly subsides, while its beneficial action in organic asthma indicates a controling power upon the circulatory apparatus, not unlike that of veratrum viride, or it may be a peculiar influence specially directed to the pneumogastric nerve.

The common people use this remedy in the shape of a strong tea, prepared by boiling the root in water in no definite proportions, or perhaps, more frequently in the form of syrup, made by boiling the root in sugar and water. We have been in the habit of using a tea of the root, a-half pound finely chipped, and simmered slowly in a quart of water for a-half or three-quarters of an hour: strain and keep in a cool place, and prepare it, fresh, every third or fourth day. Of this, one or two tablespoonsful may be taken three or four times a day. If the dose be too large, nausea will result, and probably a laxative effect upon the bowels. We seldom push it to this extreme except in cases of billiary derangement, attended with constipation. When given as an expectorant, it should be sweetened with honey or loaf sugar, and, if diarrhœa exists, a little paregoric. As a *tonic* or anti-dyspeptic, flavor with a little ginger and add a little brandy, to prevent fermentation.

An excellent formula for a syrup of this article, is the following:

R.—Button Wood Tea, strong, . . Oij.
Honey, . . . . . . . Oj.

Boil to a thick syrup and add—

Nitrate Potass., . . . . . dr. i.
Camph. Tinct. Opii, . . . . oz. i.

*Dose.*—One tablespoonful every three to four hours.

